# An Unusual Case of *Streptococcus pyogenes* Causing Ruptured Aortic Mycotic Aneurysm

**DOI:** 10.1155/2019/3035494

**Published:** 2019-07-31

**Authors:** Ali Someili, Anjali Shroff

**Affiliations:** ^1^Department of Medicine, McMaster University, 1280 Main St W, Hamilton, ON L8S 4L8, Canada; ^2^Division of Infectious Diseases, Department of Medicine, McMaster University, 1280 Main St W, Hamilton, ON L8S 4L8, Canada

## Abstract

A 70-year-old male with a complex past medical history presents with confusion and slurred speech for 24 hours. His exam was unremarkable, and his CT head was negative. Both his C-reactive protein and white blood cell count were elevated. As part of the delirium workup, blood cultures were done which grew *Streptococcus pyogenes* with no obvious source. He was treated with appropriate antibiotics. To determine the source, a white blood cell scan was done, which showed increased localization within a left-sided upper mediastinum mass. Subsequently, chest CT scan with contrast showed an acute type B aortic dissection with mycotic aneurysm. Consequently, he was taken urgently for surgical management. He completed 6 weeks of penicillin G and was discharged to a rehabilitation center. This case illustrates both a rare entity, mycotic aneurysm secondary to *Streptococcus pyogenes,* and the importance of getting an Infectious Diseases consult in the setting of an unknown source of bacteremia.

## 1. Background

Mycotic aneurysm is the infection of the arterial wall, caused by either a fungal or bacterial infection [1, 2]. In general, mycotic aneurysm is considered to be very rare, representing less than 2% of all aortic aneurysms [1]. When it does occur, it can often be a result of hematogenous dissemination or septic emboli from infective endocarditis [2]. There are no guidelines on the treatment of mycotic aneurysm, and despite the advancement of antimicrobial therapy, the mortality is still very high (10–40%) [1, 3, 4].

*Streptococcus pyogenes* is an extremely rare presentation of this, with more common organisms being *Salmonella* species and *Staphylococcus aureus* [5–8]. The mechanism of spread for *Streptococcus pyogenes* endovascular infection is often hematogenous from an infected pharynx but can be from any infected site (including skin as in on our case) [2]. To date, only 9 cases of mycotic aneurysm involving the aorta caused by *Streptococcus pyogenes* have been reported [1, 5, 9–15].

## 2. Case Presentation

A 70-year-old male presents with a past medical history of atrial fibrillation, obstructive sleep apnea, benign prostatic hyperplasia, gout, basal ganglia lacunar infarct, dyslipidemia, Gilbert's syndrome, and pituitary incidentaloma. His home medications are apixaban, pantoprazole, ezetimibe, and bisoprolol. He is a retired Stelco worker, lifelong nonsmoker, and nonalcoholic and does not use any recreational drugs. One month ago, he was admitted with mechanical fall and right leg cellulitis, for which he received cefazolin for 7 days. On this admission, he was brought in by his wife for confusion and slurred speech that has been occurring over the last 24 hours.

On examination, his vital signs were stable and he was afebrile. He was confused with no pharyngeal infection or exudate, no murmurs on cardiac exam, no adventitious sounds on respiratory examination, a soft abdomen, no joint effusions, and no visible skin lesions. There were no neurologic deficits, and pupils were equal and reactive.

## 3. Investigations

Given his symptoms, there was a high index of suspicion for stroke, and so a noncontrast head CT scan and MRI were done, both of which were negative. As part of a delirium workup, blood cultures were done.

His C-reactive protein (CRP) was 86.3 mg/l, leukocyte count was 12.2 × 1000/*μ*L, and the rest of his bloodwork was normal. On the second day of admission, one of two of his blood cultures grew *Streptococcus pyogenes*. The MRP physician looked back to his previous wound and infections in the current setting of infection but no obvious source was found.

**Figure 1:**
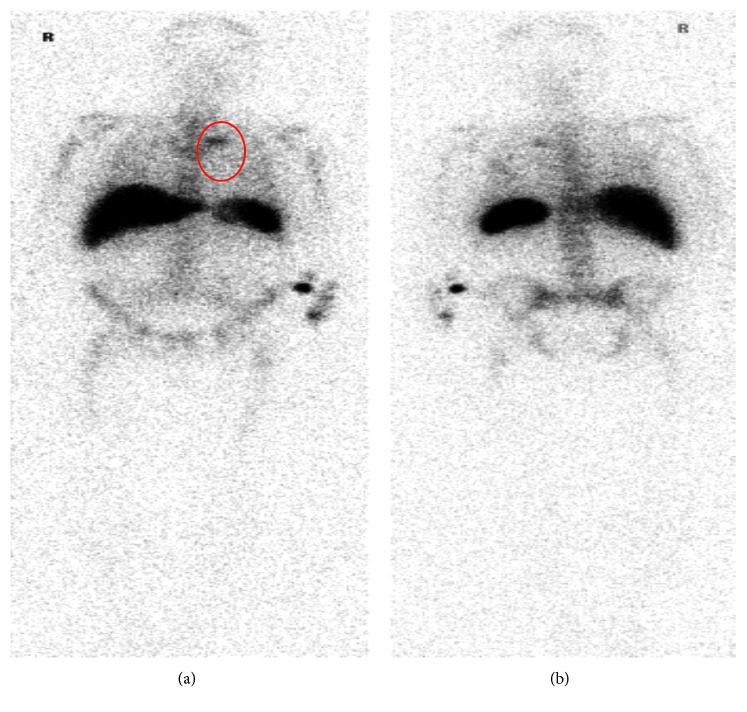
White blood cell (WBC) scan showed increased WBC localization within a left-sided upper mediastinum mass, just adjacent to the aortic arch.

**Figure 2:**
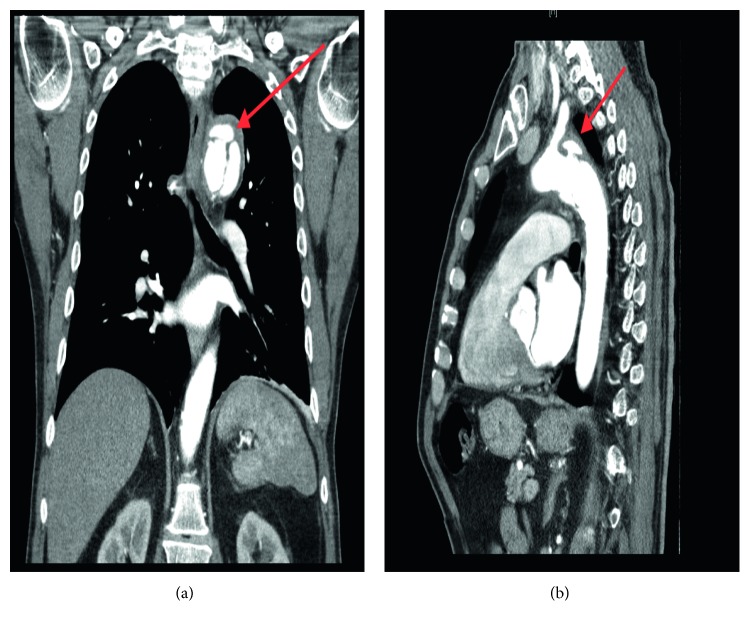
CT chest. Both (a) coronal and (b) sagittal views as indicated by red arrows showed type B aortic dissection with mycotic aneurysm.

In order to find the source, a white blood cell (WBC) scan was done which showed increased WBC localization within a left-sided upper mediastinum mass, just adjacent to the aortic arch (Figure 1). Subsequently, chest CT scan with contrast was done which showed an acute type B aortic dissection with mycotic aneurysm (Figures 2(a) and 2(b)). Echocardiography was not done, but the patient did defervesce with this antibiotic therapy, and his repeat blood cultures have been negative.

## 4. Treatment

Initially, he was started on cefazolin and then narrowed to IV penicillin G. Upon discovery of the aneurysm, he was taken urgently for surgical management via thoracic endovascular repair. He completed 6 weeks of penicillin G.

## 5. Outcome and Follow-Up

Unfortunately, postoperatively, he developed sudden paraplegia. MRI spine was done and revealed no spine infract or epidural hemorrhage. Then neurosurgery consulted and they thought that his paraplegia was likely due to spinal cord hypoperfusion secondary to the procedure, given very minimal improvement after increasing his MAP.

He completed 6 weeks of penicillin G and then was discharged to a rehabilitation center. A follow-up CT angiogram was done 3 months postoperatively which did not show any endovascular leak from the graft or any surrounding collections.

## 6. Discussion

The term mycotic aneurysm was first used by William Osler in 1885 when he described a mushroom-shaped aortic aneurysm in a patient with endocarditis [16]. The estimated incidence of mycotic aneurysm is about 0.65% to 2% of all aortic aneurysms in Western countries and reportedly higher in East Asia with estimated incidence of 3% in Taiwan of all aortic surgical procedures [17, 18]. Many pathogens have been implicated in mycotic aneurysms, with *Staphylococcus aureus* (both MSSA and MRSA) (22–28%) and *Salmonella* species (15–17%) being the most common identified organisms. The other causative organisms are *Streptococcus* species (mostly *viridians* group and pneumonia) (10%), *Enterococcus* species (11%), *E. coli* (9%), *Staphylococcus epidermidis* (8%), *Bacteroides* species (5.5%), Fungus (typically *Candida* species or *Aspergillus*) (1%), *Enterobacter* species, *Neisseria* species, *Clostridium* species, *Pseudomonas* species, and *Treponema pallidum* [5–7, 19]. Of note, the risk of endovascular infection complicating *Salmonella* bacteremia is estimated to be 9%–25% in those older than 50 years and most commonly results from seeding of atherosclerotic plaques or aneurysms [20, 21]. Up to half of the patients with infected mycotic aneurysm have sterile blood culture, and so often the organism is identified by other methods such as surgical recovery or molecular techniques. Negative cultures might be explained by the often-transient nature of this bacteremia, antibiotics being started prior to obtaining culture and by not obtaining anaerobic culture routinely [22].

The risk of mycotic aneurysm is higher in those with atherosclerosis, and arteriovenous fistula and in states of immunosuppression such as diabetes (33%), chronic renal failure (30%), chronic steroid use (16%), and chronic diseases (16%) such as rheumatoid arthritis, non-Hodgkin lymphoma, and multiple myeloma, as they can all increase the risk for hematogenous seeding [1, 23].

During the early stages of mycotic aneurysm, the symptoms are often absent or nonspecific. In a retrospective review of 33 patients with mycotic aneurysm who underwent surgery, preoperatively, signs of infection (elevated CRP and leukocytosis) were found in 79% and fever was found in only 46%. 76% of those patients had abdominal pain, and 24% already had ruptured aneurysm at the time of surgery [24]. The risk of both rupture and early rupture are higher with Gram-negative aneurysms (80% versus 10% in Gram-positive aneurysm in 2 weeks period) [24, 25].

*Streptococcus pyogenes* is associated with many different clinical presentations, including pharyngitis, skin and soft tissue infections, bacteremia, and streptococcal toxic shock syndrome including necrotizing fasciitis and glomerulonephritis but rarely endovascular infections including endocarditis or mycotic aneurysm [2, 5]. Ideally prior to endovascular repair infective endocarditis, workup should be performed but was not done, given that he was taken straight to the OR.

To date, no established guidelines to treat mycotic aneurysm exist, but the treatment in practice is a combination of antibiotics and surgery [3]. Early detection of mycotic aneurysm is extremely important as starting treatment early improves long-term survival [26]. In general, the mortality rate of mycotic aneurysm is high (23–31%) [17]. Surgical options include open resection and endovascular repair; however, the optimal surgical option remains controversial. Open debridement of mycotic aneurysm has the advantage of removing the infected tissue but is associated with high risk of mortality (13.3–40%) [27]. Endovascular repair is considered a less invasive procedure with low mortality, particularly in high risk patients, but a major disadvantage is sometimes the lack of adequate surgical debridement of the infected area. This could lead to long-term consequences due to the direct insertion of a graft into an infected field [4, 18]. There are also no clear guidelines on antibiotic choice or duration. So each case must be individually assessed based on the patient's clinical course, comorbidities, organism involved, persistence of organism, and timing of when the graft material is inserted relative to patient course. Given the complexity of this assessment, an Infectious Diseases consult can be helpful. Frequently, antibiotics are given for extended duration to treat an infected aneurysm with long-term antimicrobial suppressive therapy to prevent ongoing hematogenous seeding from the graft infection [1, 22].

In our patient, we believe his prior skin infection caused the bacteremia (perhaps transiently), which then hematogenously seeded his aorta causing the aneurysm. We treated him with only IV penicillin G, given his known *Streptococcus pyogenes* monomicrobial infection. He completed only six weeks of antibiotics, given that he had a week of therapy with negative blood cultures prior to his surgical intervention with prosthetic material.

## 7. Learning Points

- Even though *Streptococcus pyogenes* mycotic aneurysm is extremely rare, it is associated with significant mortality and morbidly
- Since there are no specific clinical presentations of mycotic aneurysm, a high index of suspicion is needed to diagnose this entity and should be considered in those with high-risk bacteremia (for example, *Staphylococcus aureus* or *Salmonella*) with no clear source or in those who are at high risk with bacteremia (for example, those with grafts in situ)
- Early detection of mycotic aneurysm is extremely important as starting treatment early improves long-term survival
- An Infectious Diseases consult should be considered to help investigate the source when the cause of bacteremia is unknown
